# Investigating endogenous µ-opioid receptors in human keratinocytes as pharmacological targets using novel fluorescent ligand

**DOI:** 10.1371/journal.pone.0188607

**Published:** 2017-12-06

**Authors:** Cheryl Leong, Christine Neumann, Srinivas Ramasamy, Bhimsen Rout, Lim Yi Wee, Mei Bigliardi-Qi, Paul L. Bigliardi

**Affiliations:** 1 Institute of Medical Biology, Agency for Science Technology & Research (A*STAR), Singapore, Singapore; 2 Institute of Chemical and Engineering Sciences, Agency for Science Technology & Research (A*STAR), Singapore, Singapore; 3 National University Hospital, Division of Rheumatology, University Medicine Cluster, Singapore, Singapore; University of Alabama at Birmingham, UNITED STATES

## Abstract

Opioids in skin function during stress response, regeneration, ageing and, particularly in regulating sensation. In chronic pruritus, topical treatment with Naltrexone changes μ-opioid receptor (μ-OR) localization to relieve itch. The molecular mechanisms behind the effects of Naltrexone on μ-OR function in reduction of itching behavior has not been studied. There is an immediate need to understand the endogenous complexity of μ-OR dynamics in normal and pathological skin conditions. Here we evaluate real-time behavior of μ-OR-Endomorphine complexes in the presence of agonist and antagonists. The μ-OR ligand Endomorphine-1 (EM) was conjugated to the fluorescent dye Tetramethylrhodamine (TAMRA) to investigate the effects of agonist and antagonists in N/TERT-1 keratinocytes. The cellular localization of the EM-TAMRA was followed through time resolved confocal microscopy and population analysis was performed by flow cytometry. The *in vitro* analyses demonstrate fast internalization and trafficking of the endogenous EM-TAMRA-μ-OR interactions in a qualitative manner. Competition with Endomorphine-1, Naltrexone and CTOP show both canonical and non-canonical effects in basal and differentiated keratinocytes. Acute and chronic treatment with Naltrexone and Endomorphine-1 increases EM-TAMRA binding to skin cells. Although Naltrexone is clinically effective in relieving itch, the mechanisms behind re-distribution of μ-ORs during clinical treatments are not known. Our study has given insight into cellular mechanisms of μ-OR ligand-receptor interactions after opioid agonist and antagonist treatments *in vitro*. These findings potentially offer opportunities in using novel treatment strategies for skin and peripheral sensory disorders.

## Introduction

Pharmacological compounds targeting opioid receptors (ORs) have been used as effective anti-nociceptive drugs. Several studies on opioid receptor functions beyond analgesia and addiction have drawn interest in skin for the past few years. ORs are G protein-coupled receptors (GPCR), which upon binding of a ligand undergo conformational changes and activate an associated inhibitory Gi/o protein. This can result in multiple responses including inhibition of adenylyl cyclase, attenuation of the influx of calcium, increase in potassium channel conductance and activation of mitogen-activated protein kinases[1].

Integrity of the skin barrier involves complex coordination of incoming signals and a controlled response at the local level as well as the interaction with the systemic immune, neural or endocrine system [2, 3]. Neuropeptides, particularly the opioidergic system, are one of the components coordinating this stress response to modulate nociceptive and inflammatory pathways in skin homeostasis and differentiation [4].

Four types of ORs, the δ-OR, κ-OR, μ-OR and nociceptin/orphanin FQ (NOP), are present in skin [5–8]. Proopiomelanocortin (POMC) and proenkephalin (PENK), the precursors of the endogenous peptide ligands endorphins and enkephalins, have been detected in human skin [7, 9–15]. Under pathological conditions such as psoriasis, atopic dermatitis or chronic wounds, dysregulation of both ORs and their corresponding ligands has been described [16–19]. Chronic pruritus could be effectively treated by topical application of the OR antagonist Naltrexone [20]. In addition, the epidermal expression of μ-OR is significantly reduced in chronic atopic dermatitis, probably due to secretion of high levels of β-endorphin in the lesional skin, which normalized under Naltrexone treatment, correlating well with the relief of itch[20, 21]. Moreover, ORs are downregulated in perilesional epidermis of chronic-non healing wounds and opioids have effects on wound healing, migration, differentiation and skin homeostasis [11, 13, 20, 22–26]. The mechanisms behind μ-OR ligand binding and receptor trafficking are crucial to explain the observed clinical efficacy of agonists and antagonists.

The lack of sufficient tools has hindered the progress to characterize endogenous ORs and the complex activity profiles of opioid ligands in skin. This has inspired to develop fluorophore-conjugated OR ligands to study endogenous cellular trafficking and opioid ligand-receptor dynamics in skin cells under different conditions. Previous reports have shown that fluorophore-conjugated OR ligands (peptide and non-peptide) can detect receptors at low density and allowed analysis of the fast kinetics of receptor ligand-interaction and flow cytometric sorting of OR-containing cell populations [27–33]. We chose Endomorphine-1 (EM), a high affinity endogenous ligand for the μ-OR, conjugated to Tetramethylrhodamine (TAMRA) to examine the cell biology of the μ-OR in keratinocytes [34]. The subcellular localization of EM-TAMRA bound μ-OR revealed a very rapid and almost complete internalization and an accumulation in the endoplasmic reticulum (ER)/Golgi network under resting conditions. Following prolonged pre-stimulation with either antagonist Naltrexone or unconjugated Endomorphine-1 the dynamics of the complex changed. The flow cytometry analyses of basal and differentiated keratinocytes reveal striking differences between the subpopulations of EM-TAMRA-positive cells.

It is essential that we understand the physiological effects of various opioid receptor agonists, antagonists and receptor-ligand dynamics in skin cells on endogenous level because of observed differences between neuronal and overexpression cell models.

## Materials and methods

### Materials

Anhydrous solvents were transferred using an oven-dried syringe. Flasks were oven dried under a stream of argon. Peptide purification was carried out using Agilent Technologies preparative HPLC system (Agilent Technologies; Santa Clara, CA, U.S.A.) on a Phenomenex Jupiter C12 reversed-phase preparative column (4 μm, Proteo 90 Å, 250 × 10 mm). The purity of the peptide was ascertained by Agilent Technologies analytical HPLC using a Phenomenex Jupiter C12 reversed-phase analytical column (4 μm, Proteo 90 Å, 150 × 4.6 mm) (Phenomenex; Torrance, CA, U.S.A.). Mass spectra were recorded on a Waters Mass-Directed LC-MS spectrometer (Waters; Milford, MA, U.S.A.) at the Institute of Chemical and Engineering Sciences, Singapore. Chemicals and anhydrous solvents used for the synthesis of the EM-TAMRA were obtained from Sigma-Aldrich (Sigma-Aldrich Pte Ltd., Singapore) and used directly, without further purification. 5(6)-Carboxytetramethylrhodamine-maleimide (TAMRA-maleimide) was purchased from Sigma Aldrich. C-terminal Cysteine-modified Endomorphine-1 was purchased from GL Biochem (Shanghai) Ltd (Shanghai, China). Keratinocyte serum-free medium (K-SFM) and supplements (Cat# 17005042) as well as TrypLE™ Express (Cat# 12605036) were from Gibco, Thermo Fisher Scientific Inc., Singapore. 1.0 M Calcium Chloride solution was purchased from Sigma Aldrich (Cat# 21114). Opioid ligands Naltrexone hydrochloride and Endomorphine-1 were obtained from Sigma Aldrich and CTOP from Tocris Bioscience (UK). All ligands were dissolved in water.

### Synthesis of TAMRA conjugated Endomorphine

In a 10 ml round bottom flask 30 mg of Endomorphine (42.0 μmol) was added to a stirred solution of a total of 18.4 mg 5(6)-TAMRA-maleimide (38.2 μmol) in 5 ml dimethylformamide (DMF). The mixture was stirred for 18 h at room temperature (RT) under an argon atmosphere. After 18 h, the reaction was monitored by diluting a drop of crude reaction mixture in acetonitrile: H_2_O (1:1) and injected in the analytical HPLC after passing through a micro filter. The DMF was evaporated under high vacuum at room temperature. The crude reaction mass was diluted with acetonitrile: H_2_O (1 ml, 1:1) and filtered through a micro filter. The filtrate was purified in preparative-HPLC.

The pure product was collected at retention time from 7.95–8.02 min (Table 1). The peak broadening was due to the 5, 6-isomers of the TAMRA dye. The purity of the HPLC fractions was ascertained by analytical HPLC. The fractions of the same purity were collected in a falcon tube and were evaporated in the lyophilizer overnight. The purity of the product (Endomorphine-TAMRA) = 95%. The weight of the product = 26.4 mg. Yield = 58%. LCMS [M+H]^+^ = 1197.03; [M+2H]^2+^ = 598.95. Peptide samples were freeze-dried in Labconco lyophilizer (Labconco, Kansas City, MO, U.S.A.) and stored at -20°C until further use. For biological experiments, EM-TAMRA was reconstituted to 1 mM concentration using water as solvent.

**Table 1:** HPLC parameters.

Time (min)	% of B	Flow rate (ml)
0	20	5
2	20	5
12	90	5
16.5	90	5
18.5	20	5
20	20	5

### Spectrofluorometry

Absorbance and fluorescence measurements were performed on a PerkinElmer EnVision 210 four Multi-label Reader (PerkinElmer Pte Ltd, Singapore). Clear bottom 96-well plates were used for absorbance and black 96-well plates for emission measurements (Costar, Corning, NY, U.S.A.). To a solution of 98 μl of keratinocyte serum-free medium (K-SFM) at a given pH, a solution of EM-TAMRA (1 mM, 2 μl) in water was added. The mixture was allowed to equilibrate for 30 min. TAMRA-Maleimide was used at a concentration of 10 mM in 2 μl water. The absorption spectra were recorded at steps of 10 nm.

For emission spectra, to a solution of 98 μl of K-SFM at a given pH, a solution of EM-TAMRA (1 mM, 2 μl) or TAMRA-Maleimide (10 mM, 2 μl) in water was added. The mixture was allowed to equilibrate for 30 min. The spectra were recorded at steps of 10 nm.

### Cell culture

N/TERT-1 human keratinocytes (Dickson *et al*., 2000) developed at Dr. J. Rheinwald’s laboratory (Harvard Medical School, Boston, MA, U.S.A.), and NHEK (normal human epidermal keratinocytes) were cultured in keratinocyte serum-free medium (K-SFM) supplemented with 0.2 ng/ml epidermal growth factor (EGF) and 25 μg/ml Bovine Pituitary Extract (BPE) (Gibco, Thermo Fisher Scientific Inc., Singapore), with 0.4mM CaCl_2_ grown to 50% confluence at 37°C in an atmosphere of 5% CO_2_. The cells were sub-cultured using TrypLE™ Express (Gibco, Thermo Fisher Scientific Inc., Singapore). For differentiation, basal N/TERT-1 cells were grown to 90% confluence, changed to K-SFM containing 1.2 mM CaCl_2_ in the absence of EGF/BPE and cultured for 7–10 days. All cells used in culture were routinely subjected to mycoplasma testing and only cells that were negative for mycoplasma were used for experiments.

### cAMP assay

N/TERT-1 keratinocytes were plated in to 96 well plates at 8000 cells/well and grown to 80% confluence. On the day of the cAMP assay the adherent cells were treated with PBS-IBMX buffer (100 μM IBMX + 0.4 mM CaCl_2_) for 30 min to inactivate phosphodiesterase. The induction buffer (PBS + 20 mM MgCl_2_) was used to dilute test compounds at different concentrations (agonist, Forskolin and TAMRA control). Cells were treated in 40 μl of induction buffer with relevant test compounds for 30 min at 37°C. 10 μl cAMP detection solution (buffer with enzyme PKA) was added to cells and incubated for 20 min. Cell lysates (50 μl) were transferred into a white-bottom 96-well plate (Greiner Bio-One GMBH, Frickenhausen, Germany). After addition of 50 μl Kinase-Glo reagent reaction was performed for 10 min before measuring luminescence using BioTek Synergy™ H1 plate reader (BioTek; Winooski, VT, U.S.A.). All the procedures were followed according to Promega cAMP-Glo™ Max Assay (Madison, WI, U.S.A.).

### Live cell imaging

Approximately 500 N/TERT-1 keratinocytes were seeded onto 4-well glass bottom chamber slides (Ibidi GmbH, Martinsried, Germany, #80427) and cultured in K-SFM supplemented with 0.2 ng/ml EGF, 25 μg/ml BPE and 0.4 mM CaCl_2_ for six days. EGF and BPE supplements were removed from the medium prior to the day of imaging. For imaging of differentiated keratinocytes cells were further grown to confluence, K-SFM containing 1.2 mM CaCl_2_ in the absence of EGF/BPE added and cells cultured for 7–10 days. For wheat germ agglutinin (WGA, Thermo Fisher Scientific Inc., Singapore) Alexa488 labeling of the membrane and endoplasmic reticulum (ER) cells were incubated in medium containing 5 μg/ml WGA conjugates for 30 min at 37°C. The cells were washed three times and fresh supplement-free K-SFM was added. Imaging before binding experiments was carried out to establish the auto-fluorescence of the cells for background adjustments. The cells were labeled at 37°C with the fluorescent-conjugated ligand at a final concentration of 200 nM and visualized by spinning disk-coupled confocal microscopy. Z-stack images were acquired using a 491 nm laser for Alexa488 and a 561 nm laser for TAMRA. The confocal unit was attached to a Nikon microscope (Singapore) with a Plan Apo 60x oil immersion objective lens (1.4 NA). Acquisition parameters were set at 20% for 561 nm laser and 5% for 491 nm laser and a motor step size of 0.1 μm was used. Images were analyzed using FIJI (ImageJ, NIH; Bethesda, MD, U.S.A.).

### Flow cytometry

Adherent basal and differentiated keratinocytes were subjected to dissociation using TrypLE (Gibco, Thermo Fisher Scientific Inc., Singapore) and washed with Phosphate Buffered Saline (PBS) before re-suspension in buffer (PBS + 1% bovine serum albumin). Ligand treatment was carried out at a density of approximately 100,000 cells per ml in buffer on ice for 30 min with the indicated concentrations before acquisition on the BD LSRFortessa™, BD FACSAria SORP™ 5-Laser Cell Sorter (BD Biosciences, San Jose, CA, U.S.A.). Signal from the TAMRA fluorophore was detected using the 561 nm excitation and PE emission filter. Samples were prepared and analyzed in triplicates. Subsequent population and data analysis was carried out using FlowJo_V10 software (FlowJo LLC, Ashland, OR, U.S.A.).

### Competition assays

N/TERT-1 keratinocytes were pre-treated with Naltrexone, CTOP or unlabeled Endomorphine-1 (all 10 μM) for 5 min. Subsequently, cells were incubated in a solution of fluorescent ligand (200 nM) plus competitor (10 μM) and immediately subjected to live cell imaging at 37°C.

For flow cytometry, N/TERT-1 cells were prepared in suspension as described above and incubated with agonist Endomorphine-1 or antagonists Naltrexone and CTOP (all 0.01–10 μM) on ice for 30 min. This was followed by the addition of 500 nM of EM-TAMRA respectively for further 30 min on ice before acquisition on the BD Fortessa™, BD FACSAria SORP™ 5-Laser Cell Sorter (BD Biosciences, San Jose, CA, U.S.A.) as described above.

### Drug treatment plan

For long-term pre-treatments with Naltrexone (10 μM) and Endomorphine-1 (10 μM), N/TERT-1 cells were incubated in the relevant media containing the drugs for two or five days. Medium was changed every 48 h of the treatment period. N/TERT-1 cells were then subjected to live cell imaging or FACS analysis as described above.

### Data and statistical analysis

All flow cytometry data are presented as mean ± SD. Experiments have been repeated four to six times (N = 4–6). In the cAMP assay each column is the average of (N = 3) data points. The change in relative light units (RLU) was calculated using the formula ΔRLU = RLU (untreated sample)–RLU (treated sample). All data were analyzed using GraphPad Prism® software, version 5.03 and subjected to ordinary One-way ANOVA using Dunnett’s multiple comparison *post hoc* test. A P value < 0.05 was considered significant. * P < 0.05; ** P < 0.01; *** P < 0.001. For chemical studies and characterization of EM-TAMRA, data were normalized using Microsoft Excel®. The raw data was obtained from measurements using PerkinElmer software EnVision ^TM^ (PerkinElmer Singapore Pte ltd).

## Results

### Synthesis, chemical and functional characterization of the Endomorphine-TAMRA conjugate

**Fig 1:**
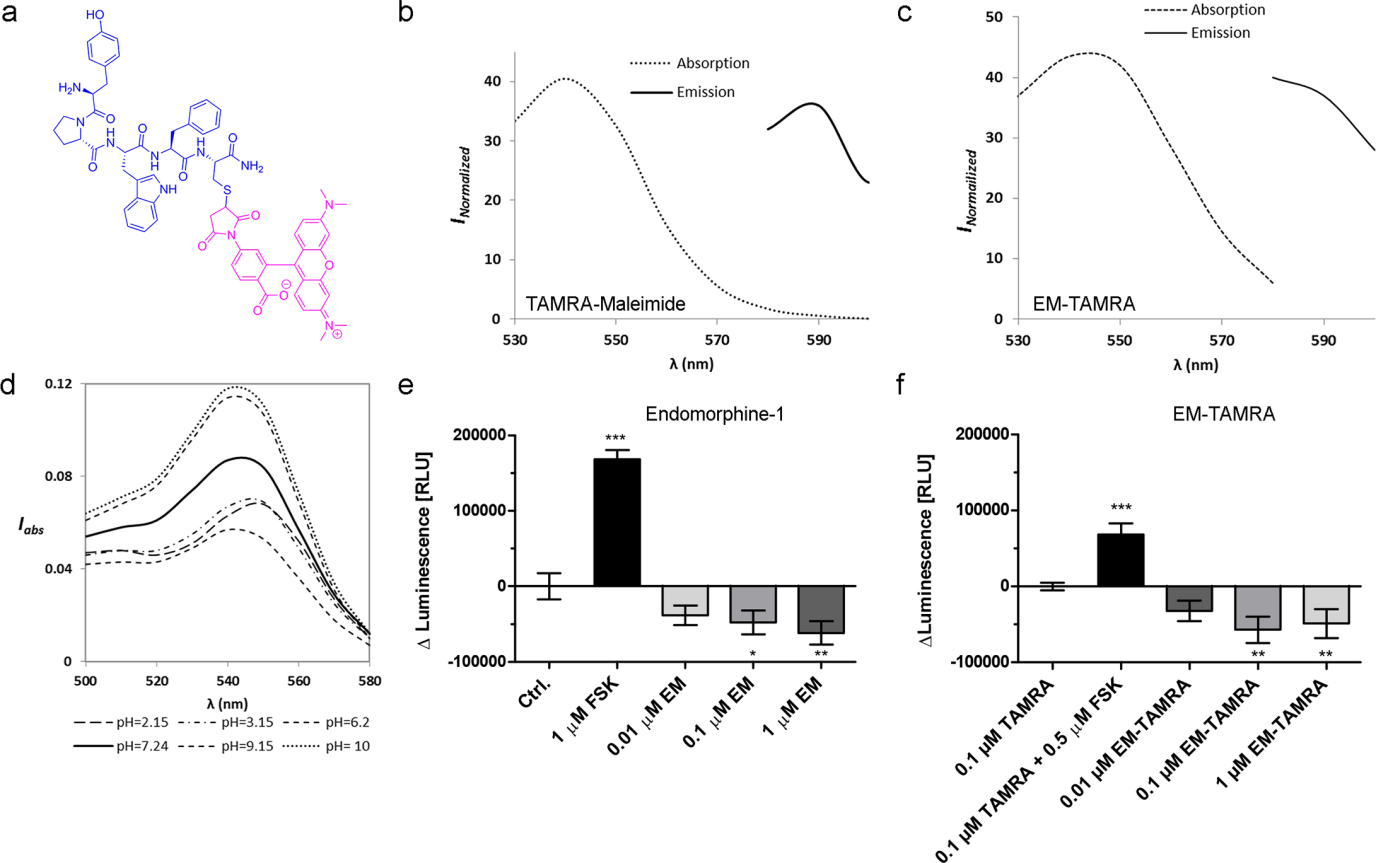
Chemical characterization of Endomorphine-TAMRA conjugates. **(A)** Chemical structure of EM-TAMRA indicated by modified Endomorphine-1 peptide in blue and TAMRA-Maleimide in magenta. Absorbance and Emission spectra of **(B)** 200 μM TAMRA-Maleimide and **(C)** 20 μM EM-TAMRA at pH 7.24. **(D)** Absorbance of 20 μM EM-TAMRA at different pH 2.15–10. I_Normalized_ = Normalized intensity; I_abs_ = Normalized absorbance.

In order to understand the endogenous receptor dynamics and regulation of the μ-OR in N/TERT-1 keratinocytes, we generated fluorophore-conjugated Endomorphine-1 (EM-TAMRA) (Fig 1A and S1 Fig). A method previously described was adapted to synthesize the compound [30]. Modified Endomorphine-1 with an additional cysteine at the C-terminus, provides a thiol group, which was conjugated to the maleimide of TAMRA by an addition reaction (S1 Fig). The reaction was conducted in the polar solvent dimethylformamide to avoid the use of any base. The yield of the reaction is moderate and the HPLC purity of the EM-TAMRA obtained was high (95%) (S2A Fig). The conjugated product was characterized using ESI mass spectroscopy to determine the molecular mass of EM-TAMRA. The molecular ion peak is at 1197.03 and its double charge at 598.95 confirms the formation of conjugated product (EM-TAMRA) (S2B Fig).

The absorption and emission spectra of both TAMRA-Maleimide and EM-TAMRA at pH 7.24 were determined (Fig 1B and 1C). A slight shift in absorption maxima from 540 nm to a longer wavelength of 550 nm was observed after conjugation of EM to TAMRA-Maleimide. The emission spectra collected between 580 nm and 600 nm wavelengths show minimal change of the emission maxima after conjugation (Fig 1B and 1C).

The absorption spectra of EM-TAMRA measured in K-SFM culture medium slightly change over the range of pH 2–10 (Fig 1D). With increasing pH from acidic to basic environment, the absorption maxima peak decreased. The maximum absorption intensities of EM-TAMRA were observed between pH 9–10 and moderate absorption intensity was found at physiological pH 7.24. However, the maximum emission intensities of EM-TAMRA at a particular concentration, measured at two different wavelengths were observed at 580 nm, pH 7.24 (S3 Fig). The stability of linkage between thiol group of the short cysteine-modified Endomorphine peptide and maleimide of the hydrophobic dye TAMRA was analyzed at different time intervals. The HPLC detector at 254 nm and 570 nm was used at physiological pH 7.24 to show the purity of EM-TAMRA. It was observed that HPLC purity remains stable for 90 min and no extra impurity peaks were observed at these wavelengths (for detailed HPLC see supporting information, S4A–S4C Fig).

Biological functionality of the EM-TAMRA conjugate was tested by detection of changes in the cAMP level in N/TERT-1 keratinocytes upon ligand treatment. Similar to Endomorphine-1, EM-TAMRA decreased the accumulation of cAMP. In contrast, treatment with TAMRA dye alone did not affect cAMP levels and the positive control Forskolin increased cAMP levels in keratinocytes. This indicates that EM-TAMRA is biologically active (S5A and S5B Fig).

### Keratinocytes specifically bind and internalize the endomorphine-TAMRA conjugate *in vitro*

**Fig 2:**
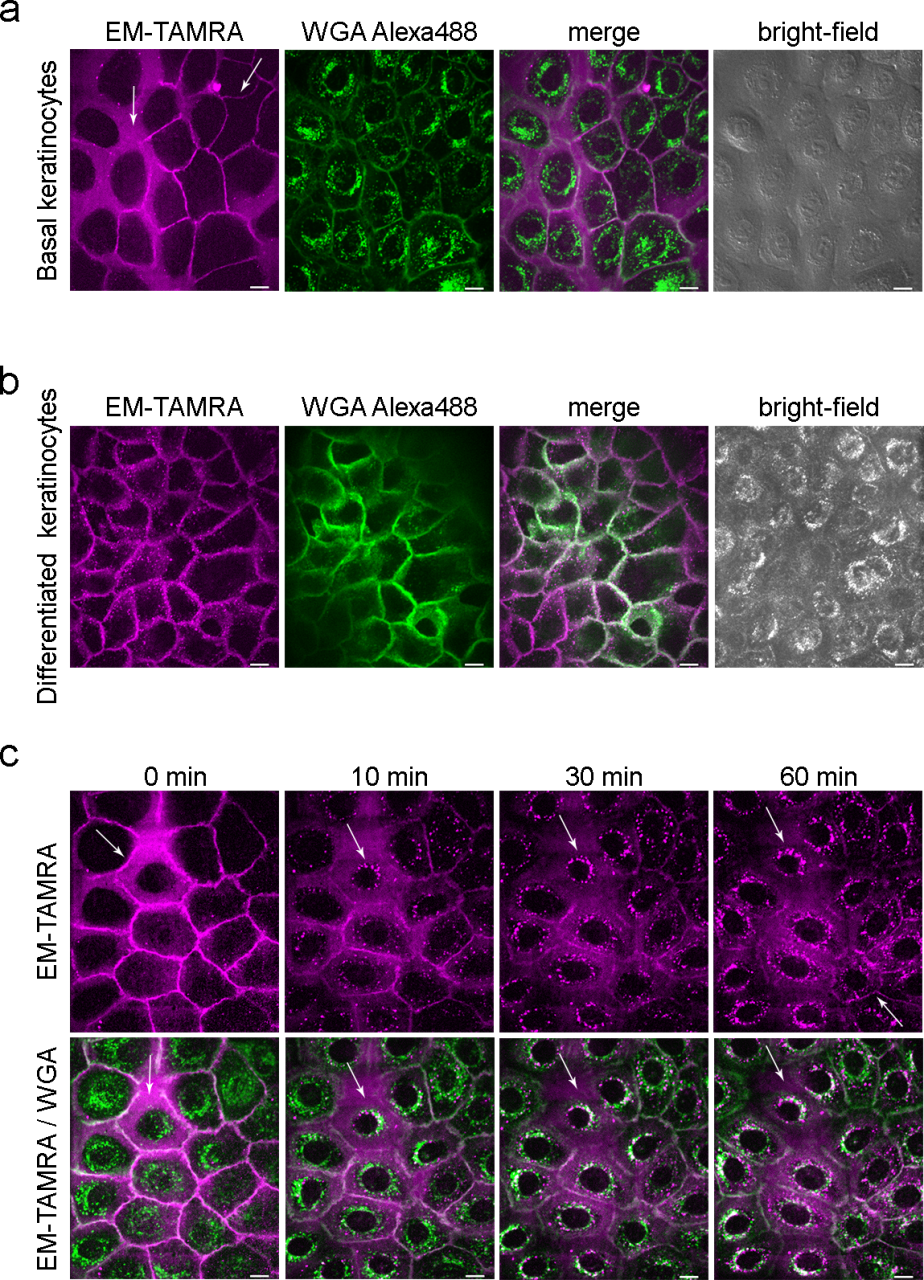
Keratinocytes bind and internalize Endomorphine-TAMRA conjugate. Keratinocytes were subjected to live cell imaging using confocal microscopy at 37°C. After labeling membranes and endoplasmic reticulum with WGA (green), 200 nM EM-TAMRA was added to the cells and image acquisition started immediately. (A) Representative images of a colony of basal N/TERT-1 cells show strong membrane binding of EM-TAMRA (magenta) in some areas and more diffuse staining in other regions (arrows). (B) After seven days of differentiation more pronounced membrane staining is visible. Small puncta appear close to the membrane as a result of the fast internalization process. (C) Time course of EM-TAMRA internalization in basal keratinocytes. At the start strong membrane binding is visible. After 10 min the majority of EM-TAMRA has been internalized and appears in the ER/Golgi perinuclear network as indicated by WGA co-labeling in white (arrows). Some membrane staining is retained after 60 min incubation. The majority of EM-TAMRA accumulates intracellular (arrow in EM-TAMRA 60 min). Images displayed are SUM-projections of ten slices from a Z-stack image with 0.1 μM step size. Co-localization of EM-TAMRA and WGA appears white. Scale bar represents 10 μm. WGA = wheat germ agglutinin.

Live cell binding of EM-TAMRA to the adherent keratinocyte cell line N/TERT-1 was visualized using a confocal microscope. At a concentration of 200 nM, EM-TAMRA selectively stained the membrane of keratinocytes as observed by co-staining with the membrane marker wheat germ agglutinin (WGA). A similar staining pattern appeared in both basal and differentiated keratinocytes (Fig 2A and 2B). Binding was observed within 1 min after addition of ligand. Prolonged incubation with the EM-TAMRA showed internalization within 10–30 min (Fig 2C). Membrane binding was lost and intracellular puncta appeared close to the membrane and were transported towards the perinuclear region where they accumulated within 60 min of incubation. WGA co-staining confirmed co-localization of internalized compound with the endoplasmic reticulum (Fig 2C). In contrast to the EM-TAMRA conjugate, no binding and internalization was observed for TAMRA dye alone (S6 Fig). In two-dimensional differentiation, keratinocytes pseudo-stratify and form several layers of cells. In the differentiated cell layers this results in uneven labeling of both EM-TAMRA and co-stain marker WGA individually. The live cell observation of differentiated populations of keratinocytes using confocal microscopy was technically not feasible (Fig 2B and S7 Fig).

### Endomorphine-TAMRA enables the detection of distinct positive populations in both basal and differentiated N/TERT-1 keratinocytes

**Fig 3:**
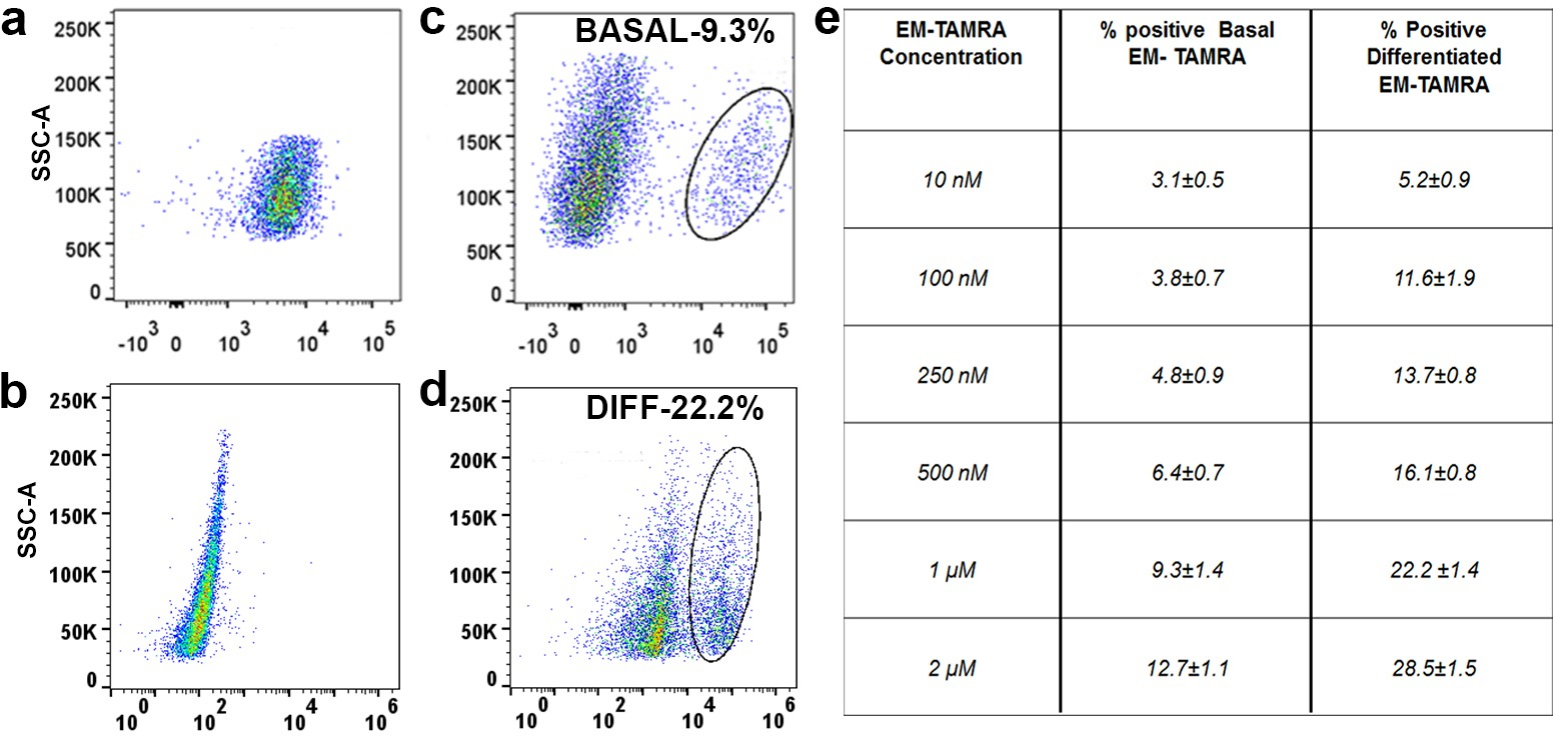
Flow cytometry analyses of keratinocyte basal and differentiated populations bound by TAMRA and EM-TAMRA. EM-TAMRA labeling of both basal and differentiated keratinocytes were performed in 4°C at a dose of 500 nM. The labeled cells were subjected to population based flow cytometry analysis to study basal keratinocytes (A and C) and differentiated keratinocytes (B and D) using TAMRA dye alone (A and B) and EM-TAMRA (C and D). Representative images in (C) and (D) show specific separation of sub-population in EM-TAMRA labeled basal and differentiated keratinocytes distinct from TAMRA dye alone. Specific population was observed with respective percentages for both basal (8.3%) and differentiated (22.2%) cell types as represented in Table E. Data shown are calculated from six independent biological replicates (N = 6) and represent mean ± SD for respective concentrations (10 nM – 2 μM).

The addition of 10 nM-2 μM EM-TAMRA presented a distinct population ranging from 3–14% for basal cells and 6–28% for differentiated cells (Fig 3A–3E). This shows substantial difference in labeling between basal and differentiated cells. Cell staining with unconjugated TAMRA fluorophore (Fig 3A and 3B) only showed a single homogenous population, in comparison to EM-TAMRA (Fig 3C and 3D). Experiments using normal human epidermal keratinocytes (NHEKs) showed similar cell staining and scatter profiles to that of basal N/TERT-1 keratinocytes (S8 Fig). However, for the purpose of clear population visualization and separation, concentrations of up to 500 nM were used for evaluation in flow cytometry experiments.

### Competition assays with agonist/antagonist show selectivity towards binding of Endomorphine-TAMRA

**Fig 4:**
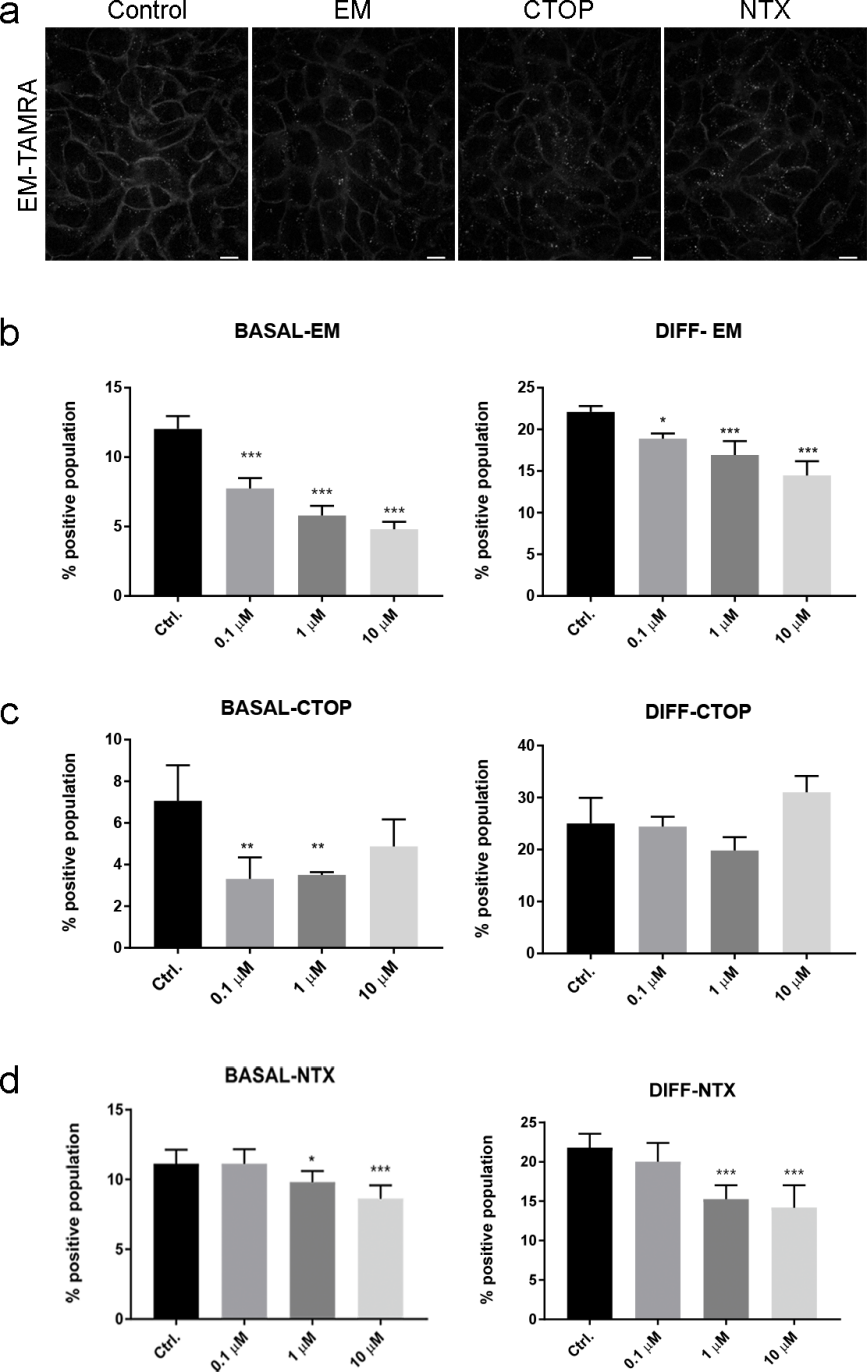
Competition with μ-OR ligands shows specificity of EM-TAMRA binding and differences between basal and differentiated keratinocyte populations. (A) For live cell imaging, cells were pre-incubated with 10 μM competitor for five min before addition of EM-TAMRA (200 nM) in the presence of 10 μM competitor at 37°C. As compared to the control, Endomorphine-1 was more effective in competing for membrane binding with EM-TAMRA than CTOP and Naltrexone in basal N/TERT-1 cells. Scale bar represents 10 μm. (B) Flow cytometry analysis after 30 min pre-treatment with ligands on ice show significant reduction in EM-TAMRA positive populations during competition with Endomorphine-1 at low (100 nM) as well as high (10 μM) concentrations in both basal and differentiated keratinocytes. (C) CTOP (100 nM and 1 μM) was able to block EM-TAMRA binding in basal N/TERT-1 but not in differentiated cells. (D) Naltrexone shows competition at higher concentrations of 1 μM to 10 μM but not at low concentration of 100 nM. Data are represented as mean ± SD from four replicate experiments (N = 4) and were subjected to ordinary One-way ANOVA using Dunnett’s multiple comparison *post hoc* test. * P < 0.05; ** P < 0.01; *** P < 0.001. EM = Endomorphine-1; NTX = Naltrexone.

A pronounced inhibition of binding and internalization of EM-TAMRA was observed in competition assays with unlabeled Endomorphine-1. The presence of the opioid antagonists Naltrexone and CTOP reduced the binding of fluorescent ligand. Internalized fluorescent puncta became distinguishable within five min of incubation in the presence of antagonists. The cells incubated with antagonists showed that the EM-TAMRA signal was distributed in the intracellular compartments while maintaining a weaker membrane binding signal over a period of 30 min (Fig 4A).

Similar results were observed in flow cytometry analysis. To determine the specificity of the observed EM-TAMRA positive populations, a competition assay was carried out by pre-incubating the cells with unconjugated ligand before addition of EM-TAMRA. Unlabeled Endomorphine-1 was able to block EM-TAMRA binding in a concentration dependent manner, in both basal and differentiated keratinocytes (Fig 4B). The competition with Endomorphine-1 (EM) was effective even at lower dose (50–100 nM) in both basal and differentiated cell populations (S9 Fig). The μ-OR specific antagonist CTOP was able to inhibit EM-TAMRA binding in basal keratinocytes at concentrations of 0.1–1 μM but had no effect on differentiated cells (Fig 4C). The classical opioid antagonist Naltrexone was observed to cause a partial reduction of the percentage positive population at specific concentrations ranging from 1–10 μM for basal and differentiated N/TERT-1 labeled with EM-TAMRA (Fig 4D).

### Effect of acute and chronic μ-OR agonist/antagonist exposure in keratinocytes

**Fig 5:**
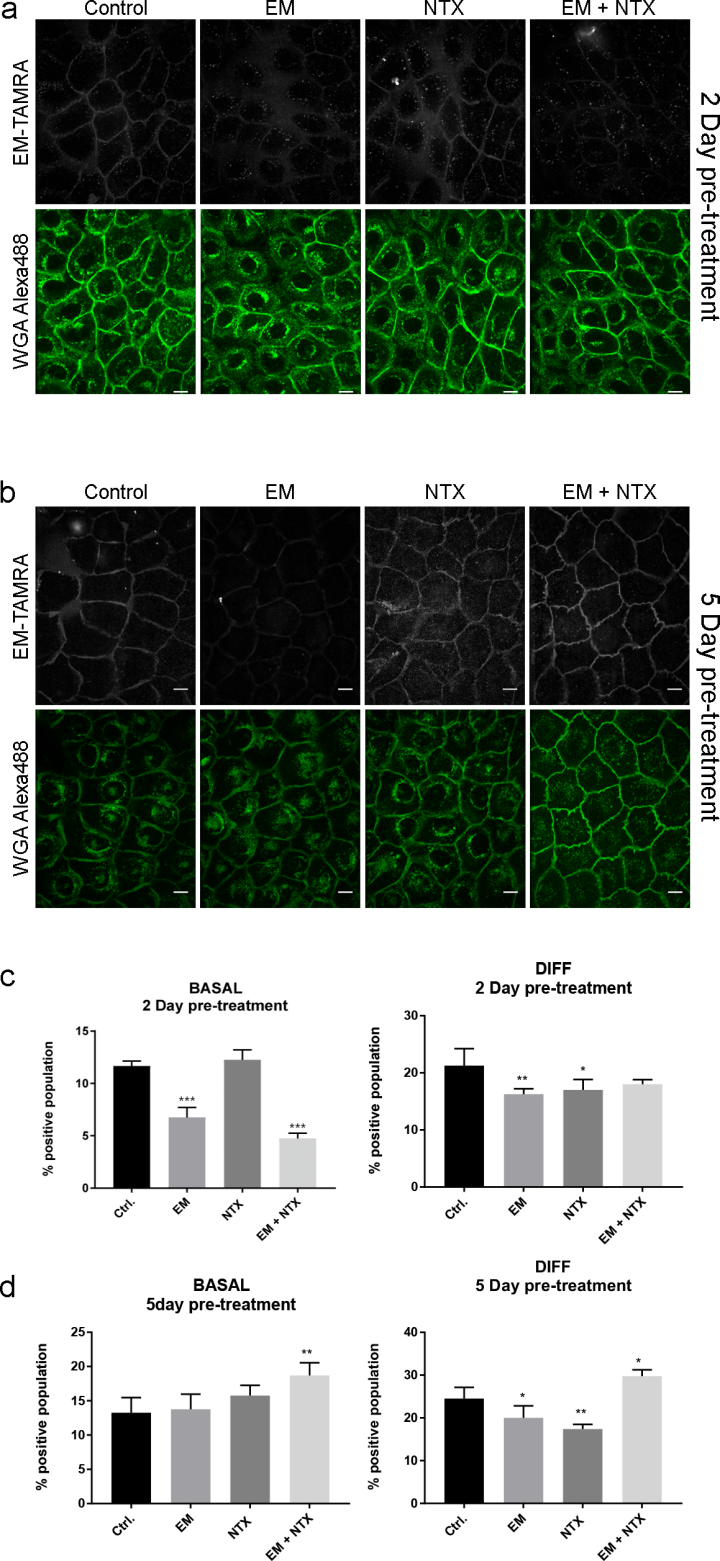
Acute and chronic exposure of keratinocytes to opioid antagonist Naltrexone and agonist Endomorphine-1 affect cell membrane localization of μ-OR and enhances binding of EM-TAMRA. (A) Live cell imaging shows reduced binding of EM-TAMRA (grey) in two day Endomorphine-1 and Endomorphie-1 + Naltrexone double pre-treated cells. Subtle differences are observed in Naltrexone treated cells. Scale bar represents 10μm. (B) In chronic, five day pre-treated cells a clear reduction of EM-TAMRA binding is observed in Endomorphine-1 and Naltrexone treated cells. The double treatment with Endomorphine-1 and Naltrexone results in increased binding of EM-TAMRA to keratinocytes. Scale bar represents 10 μm. (C) Flow cytometry analyses reveal reduction of EM-TAMRA binding in Endomorphine-1 and Naltrexone + Endomorphine-1 double treated basal cells after two day incubation similar to live cell imaging data from basal cells. In differentiated keratinocytes all treatments led to a reduction of EM-TAMRA positive populations. Only Endomorphine-1 and Naltrexone individual treatments display statistically significant reductions. (D) Chronic treatment with Naltrexone + Endomorphine-1 combination results in increased binding of EM-TAMRA in basal keratinocytes. In differentiated cells single compound treatment reduces EM-TAMRA positive populations but the double treatment with Naltrexone + Endomorphine-1 increases binding. Data are represented as mean ± SD from four replicate experiments (N = 4) and were subjected to ordinary One-way ANOVA using Dunnett’s multiple comparison *post hoc* test. * P < 0.05; ** P < 0.01; *** P < 0.001. WGA = Wheat germ agglutinin, EM = Endomorphine-1; NTX = Naltrexone.

Topical drug treatment with Naltrexone in chronic pruritus shows remarkable change in μ-OR localization and itch sensation in patients [20]. In order to mimic the pathological environment of high levels of OR agonist β-endorphin and low levels of OR in the membrane found in affected patients, we exposed basal and differentiated keratinocytes to acute and chronic naltrexone treatment and monitored the effect on the binding and internalization of EM–TAMRA. Keratinocytes were incubated individually with antagonist Naltrexone or agonist Endomorphine-1 and in combination (Naltrexone + Endomorphine-1) at concentrations of 10 μM. After a period of two or five days, equivalent to acute or chronic conditions, skin cells were subjected to live cell imaging upon addition of EM-TAMRA and observed for 1 h. Reduced membrane binding and internalization was observed in basal cells after two days of pre-treatment with Endomorphine-1 alone or Naltrexone + Endomorphine-1 co-treatment (Fig 5A). In the five day treatment, a decrease of EM-TAMRA label both in membrane and cytosol was observed in the Endomprhine-1 or the Naltrexone single treatment. The co-treatment with both Naltrexone and Endomorphine-1 had an increased labeling of EM-TAMRA in the membrane and the cytosol (Fig 5B). Using flow cytometry analysis, we found that two day (acute) pre-treatment with Endomorphine-1 or co-treatment with Naltrexone in basal cells reduces EM-TAMRA labeling (Fig 5C). In differentiated keratinocytes co-treatment with Naltrexone + Endomorphine-1 did not decrease EM-TAMRA positive populations except Endomorphine-1 and Naltrexone individual treatments that display statistical significance (Fig 5C). Chronic treatment of basal cells for five days shows that only co-treatment of Naltrexone + Endomorphine-1 increases EM-TAMRA positive cells, while individual treatments did not show any difference in EM-TAMRA labeling (Fig 5D). In chronic treated differentiated keratinocytes, co-treatment with Naltrexone + Endomorphine-1 shows an increased EM-TAMRA positive population in comparison to reduced labeling in individual treatments (Fig 5D).

## Discussion

Previous studies demonstrated a reduction of epidermal OR expression in chronic wounds, chronic pruritic dermatoses, and psoriasis [20, 21, 24]. While the endogenous ligand β-endorphin is highly expressed in these skin conditions the dysregulation of OR expression might increase disease symptoms such as pruritus. The opioid antagonists Naloxone, Naltrexone and κ-OR agonists are used systemically and topically for the treatment of various types of chronic pruritus, e.g. in liver diseases or chronic dermatoses [8, 35–41]. We reported a potential role for Naltrexone in relieving the itch sensation by increasing the functional presence of μ-OR in skin keratinocytes within two weeks of topical treatment [20]. The complex cellular basis and mechanism of Naltrexone responsiveness to restore homeostasis in chronic pruritic skin is not clearly understood. One suggestion is that Naltrexone potentially acts as a pharmacological chaperone on cellular level, leading to an increase of μ-OR cell membrane localization [42–44]. In different experimental itch models in animals, overexpression cell models, using non-skin, primary neuron models or in fixed tissue, researchers investigated the effectiveness and pharmacological response of antipruritic compounds [45, 46]. However, there are no studies that understand real-time dynamics of opioid receptor binding and internalization in response to agonist and antagonist treatment in cultured skin cells. The pharmacological response behind agonist and antagonist treatments and their role in opioid ligand-receptor activity in skin also remain to be studied in detail. Our study uses fluorescent opioid peptides to observe endogenous ligand-receptor trafficking in normal skin keratinocytes. We chose Endomorphine-1 because of its reported high affinity and rapid internalization upon binding to the μ-OR [47, 48]. The N-terminus of Endomorphine-1 contains the message necessary for μ-OR recognition [49]. Therefore, the fluorescent dye was conjugated to the C-terminus of cysteine-modified Endomorphine-1 to minimize the loss of biological activity. Previously, Arttamangkul *et al*. synthesized an Alexa Fluor 488 conjugated Endomorphine-1 peptide. This study reported that the fluorescent dye Alexa Fluor 488, which bears a negative (SO_3_^2-^) charge, might contribute to the non-effective internalization of the peptide-fluorophore conjugate. To circumvent this issue they had used BTR conjugate that mimicked native internalization pattern of the ligand [30]. We used TAMRA which has similar range of properties, a photo-stable fluorophore with absorption at 550 nm, which has two aromatic nitrogen. The TAMRA-maleimide has an α β-unsaturated double bond present on the maleimide backbone and is therefore a very good nucleophile receptor to react with thiol or amine groups. The cysteine amino acid at the C-terminus of the modified Endomorphine-1 contains a thiol moiety, which reacts with this α, β-unsaturated double bond of maleimide and provides EM-TAMRA conjugate in moderate yield (S2 Fig).

Keeping in mind that different cellular compartments have different pH, the dynamic stability of the EM-TAMRA conjugate *in vitro* is of high importance. The stability tests of EM-TAMRA in a range of pH 2–10 buffer conditions suggest that EM-TAMRA is stable between pH 2–10 without any detectable impurity formation. This pH stability also excludes the dissociation of the cysteine-maleimide bond and shows that the EM-TAMRA conjugate has acceptable durability under our experimental conditions (S4 Fig). The emission peaks of TAMRA-maleimide broaden upon conjugation to Endomorphine-1. This is probably due to the slight reduction in the rotation of the C-C bond between the xanthene and phenyl part of TAMRA (Fig 1B and 1C). The maximum emission intensity at the same concentration suggested that the conjugate is suitable and stable for the experimental conditions during live cell imaging and flow cytometry analysis (S3 Fig).

Activation of μ-OR by Endomorphine-1 decreases intracellular cAMP levels [50]. Addition of EM-TAMRA to skin cells also reduces cAMP levels indicating that the conjugate has biological activity similar to unconjugated Endomorphine-1 (S5 Fig). This provides the opportunity to use EM-TAMRA and investigate endogenous receptor-ligand dynamics in skin cells.

The membrane labeling of EM-TAMRA reflects a uniform staining pattern of the endogenous receptor, which is different from previous reports where receptor clustering is observed in fixed samples [51]. Other over-expression studies using Dermorphine-BODIPY Texas Red-conjugates suggest local clustering or dimerization of μ-OR receptor, which can influence endocytosis [52]. Using live cell imaging we observe that the ligand stays in the membrane for a very short period of one to five minutes, but our systems or tools do not capture clustering of μ-ORs at high enough resolution. Likewise, internalization and punctate distribution of μ-OR-ligand complexes have been observed in primary neurons using fluorescent ligand conjugates and were reported to be distinct from de- or re-sensitization mechanisms [53]. The time dependent internalization of μ-OR within 10 min upon addition of etorphine and DAMGO observed in non-skin models was similar to the time dependent internalization in EM-TAMRA using skin keratinocytes [48, 54, 55]. This suggests that TAMRA conjugation did not affect the biological function of the ligand (S6 Fig). EM-TAMRA internalizes, localizes to the perinuclear ER/Golgi network and can be detected as vesicular or punctate pattern for 60–120 min as proven by WGA co-labeling. EM-TAMRA dynamics indicate a μ-OR-specific endocytosis pathway because no membrane binding and internalization was observed with the TAMRA dye alone. There is no or weak residual labeling of the membrane after internalization of EM-TAMRA suggesting it is biologically active (Fig 2C).

Keratinocytes exist as heterogeneous population, which affects the labeling of EM-TAMRA and its distribution pattern. The clonal variability and environmental parameters in keratinocyte cultures influence the expression profile of opioid receptors. Adherent keratinocytes take up EM-TAMRA as observed during live cell imaging in contrast to labeling of a cell suspension for subpopulation analysis by flow cytometry. It should also be considered that imaging experiments were performed at 37°C allowing fast receptor regulation and repositioning, while flow cytometry studies were performed at 4°C, delaying or stabilizing receptor movements. This reflects the different states of the cells under adherent and suspension conditions. The observed positive labeling of EM-TAMRA in comparison to TAMRA in both basal and differentiated cells suggests that the specific sub-population shift is due to binding of Endmorphine and not TAMRA in flow analysis (Fig 3A–3D).

The increase of EM-TAMRA staining in differentiated compared to basal immortalized N/TERT-1 keratinocytes correlates well with an observed increase of μ-OR mRNA expression in the differentiated layers of human epidermis [5].

Lack of EM-TAMRA labeling in the membrane and perinuclear vesicles during competition studies with unconjugated Endomorphine-1 suggest μ-OR- specific ligand binding and internalization (Fig 4A). The flow cytometry analysis uses a specific dose range of Endomoprhin-1 at concentrations 10 nM to 10 μM to reduce the EM-TAMRA label and doses from 50–100 nM are sufficient to compete away the labeling (Fig 4B). These data align well with previous reports of the high μ-OR affinity peptide Endomorphine-1 [34, 47].

Interestingly, our imaging studies show that pan-opioid antagonists Naltrexone and μ-OR specific antagonist CTOP are partially able to block internalization of EM-TAMRA, however unconjugated Endomorphine-1 blocks membrane and intracellular labeling more effectively. This suggests that EM-TAMRA behaves similar to Endomorphine-1 in its affinity to μ-OR in contrast to Naltrexone and CTOP. Recent studies also suggest that Endomorphine-1 may have non-opioid targets in living cells as demonstrated by antagonist and inhibitor studies [56]. However, the difference in efficacies of Naltrexone, CTOP and unconjugated Endomorphine-1 in competition assays needs more detailed investigation using radio-ligand binding assays. In flow cytometry analysis, CTOP shows partial inhibition in basal but not differentiated cell populations (Fig 4C). This indicates that the EM-TAMRA bound endogenous μ-OR in keratinocytes does not respond to classical pharmacological OR antagonists as opposed to isolated membrane binding assays or animal models [50, 57]. The physiological environment in basal and differentiated keratinocyte cultures could potentially affect the affinity of CTOP to the endogenous μ-OR since studies related to sodium ion concentration show lower affinity of CTOP [58, 59].

In order to address the long-term effect of opioid agonist and antagonist, similar to the clinical setting of pruritus, basal and differentiated keratinocytes were exposed to prolonged Endomorphine-1 treatment along with Naltrexone. During five days of ligand incubation, an increase of EM-TAMRA binding on the cells was observed in the Endomorphine + Naltrexone double treatment group in both basal and differentiated keratinocytes using live cell imaging and flow cytometry analysis (Fig 5A–5D). Long term treatment with antagonist Naltrexone stabilizes the μ-OR in the membrane probably due to chaperone specific effects on maturation and recycling[44]. As a result more binding of opioid receptor ligand and finally more μ-OR efficacy and function is observed in skin cells. This is one of the probable reasons why we observe an increased expression of epidermal μ-OR leading to reduced itch sensation as seen in chronic pruritic dermatoses [20]. Under different environmental and prevailing pathological condition dysregulation of the opioid system has been observed leading to non-classical local drug responses [20, 21, 60].

## Conclusion

Our study has given specific understanding on cultured basal and differentiated human keratinocyte behavior on specific μ-OR-ligand dynamics. Although this study on healthy cultured skin cells will not be a direct correlate to pathological skin cells, efforts to culture and immortalize pruritus cell types may be challenging. The fluorophore conjugate EM-TAMRA has given us an opportunity to understand how drug treatments can affect normal cultured skin cells. However, it remains to be understood how opioid antagonists and agonists exert control in normal cellular behavior to restore skin homeostasis. The functional dynamics of agonist and antagonist controlled opioid receptor signaling in the brain and skin do emphasize that physiological effects can be canonical or non-canonical dependent on specific contexts. The treatment strategies using opioid drugs require careful consideration by physicians.

## Supporting information

S1 Fig Schematic representation of addition reaction of modified Endomorphine-1 with TAMRA-Maleimide. (TIF)

S2 Fig HPLC purity profile of EM-TAMRA and ESI mass spectrum of EM-TAMRA. (A) EM-TAMRA was purified using a Jupiter C12 Proteo 90Å RP-HPLC preparative column (10 mm x 250 mm x 4 μm), detector wavelength (220 nm, 546 nm) and following solvent gradients, solvent A (100% H_2_O + 0.1% TFA) and solvent B (100% acetonitrile + 0.1% TFA). The purity of each fraction were analysed using an analytical HPLC with a Jupiter C12 Proteo 90Å RP-HPLC column (4.6 mm x 150 mm x 4 μm), detector wavelength (220 nm, 546 nm) and following solvent gradients, solvent A (100% H_2_O + 0.1% TFA) and solvent B (100% acetonitrile + 0.1% TFA).
(B) ESI mass of EM-TAMRA was taken using solvent A (100% H_2_O + 0.1% Formic acid, solvent B (100% acetonitrile + 0.1% formic acid). It was run in isocratic 80:20 (B:A) for two minutes without passing a column. The capillary voltage used during measurement was 3.50 kV.
(TIF)

S3 Fig Histogram of EM-TAMRA emission at different pH. The emission spectra of EM-TAMRA (20 μM) was measured at 580 nm and 590 nm wavelengths for six different pH of K-SFM buffer. λ_exc_ = 560 nm.
(TIF)

S4 Fig Stability study of EM-TAMRA using HPLC at different pH. Stability of EM-TAMRA at pH 3.48 (B) and pH 8 (C) in keratinocyte serum-free medium (K-SFM) were studied by analytical HPLC incubating EM-TAMRA for 90 minutes. HPLC of samples were measured using 570 nm to investigate dissociation products. EM-TAMRA is stable under both acidic pH 3.48 (B) and basic pH 8 (C) condition as compared to no K-SFM buffer (A).
(TIF)

S5 Fig Functional characterization of EM-TAMRA. N/TERT-1 keratinocytes were plated in to 96-well plates at 8000 cells/well and grown to 80% confluence. On the day of the cAMP assay the adherent cells were treated with PBS-IBMX buffer (100 μM IBMX + 0.4 mM CaCl_2_) for 30 min to inactivate phosphodiesterase. The induction buffer (PBS + 20 mM MgCl_2_) was used to dilute test compounds at different concentrations (agonist, Forskolin and TAMRA control). Cells were treated in 40 μl of induction buffer with relevant test compounds for 30 min at 37°C. 10 μl cAMP detection solution (buffer with enzyme PKA) was added to cells and incubated for 20 min. Cell lysates (50 μl) were transferred into a white-bottom 96-well plate (Greiner Bio-One GMBH, Frickenhausen, Germany). After addition of 50 μl Kinase-Glo reagent reaction was performed for 10 min before measuring luminescence using BioTek Synergy™ H1 plate reader (BioTek; Winooski, VT, U.S.A.). All the procedures were followed according to Promega cAMP-Glo™ Max Assay (Madison, WI, U.S.A.). Inhibition of cAMP production upon opioid receptor activation by Endomorphine-1 or the EM-TAMRA conjugate was analyzed.
(A) cAMP level relative to untreated control in Forskolin stimulated or Endomorphine-1 (0.01 μM– 1 μM) treated N/TERT-1 keratinocytes. (B) cAMP level in N/TERT-1 keratinocytes normalized to TAMRA control treated samples. Forskolin stimulation was done in the presence of TAMRA to exclude influence of the dye on the assay reading. EM-TAMRA was added in concentration from 0.01 μM to 1 μM. Data from one representative experiment are represented as mean ± SD from three technical replicates. Ctrl. = untreated control; FSK = Forskolin; EM = Endomorphine-1; RLU = Relative Light Units.
(TIF)

S6 Fig Unconjugated fluorescent dye TAMRA-Maleimide does not bind to N/TERT-1 keratinocytes. N/TERT-1 keratinocyte membrane and endoplasmic reticulum was labelled for 30 min at 37°C with 5 μg/ml Wheat Germ Agglutinin (WGA Alexa Fluor 488, Thermo Fisher Scientific Inc., Singapore). The cells were washed three times and fresh supplement-free K-SFM was added. TAMRA-Maleimide was diluted in K-SFM containing 0.4 mM CaCl_2_ in the absence of EGF/BPE. Imaging before binding experiments was carried out to establish the auto-fluorescence of the cells for background adjustments. TAMRA was added at a final concentration of 200 nM and cells visualized by spinning disk-coupled confocal microscopy. Z-stack images were acquired using a 491 nm laser for Alexa488 and 561 nm lasers for TAMRA. Acquisition parameters were set at 20% for 561 nm laser and 5% for 491 nm laser and a motor step size of 0.1 μm was used. Images were analysed using FIJI (ImageJ, NIH; Bethesda, MD, U.S.A.).
Weak non-specific staining of keratinocytes by TAMRA can be observed due to the interaction of the dye with lipids of the cell membrane. The staining intensity and pattern does not reflect the staining observed for EM-TAMRA. No internalisation of TAMRA is seen after prolonged incubation over 2 h. EM-TAMRA keratinocyte labelling is therefore caused by the specific interaction of EM with μ-OR cell surface receptors and receptor-mediated internalisation.
(TIF)

S7 Fig Uneven labelling of differentiated N/TERT-1 cells due to pseudo-stratification. N/TERT-1 keratinocytes were differentiated for ten days and then subjected to 5 μg/ml WGA Alexa Fluor 488 staining for 30 min at 37°C. The cells were washed three times and fresh supplement-free K-SFM was added. EM-TAMRA-Maleimide was diluted in K-SFM containing 0.4 mM CaCl_2_ in the absence of EGF/BPE. Imaging before EM-TAMRA labelling experiments was carried out to establish the auto-fluorescence of the cells for background adjustments. EM-TAMRA was added at a final concentration of 200 nM and cells visualized by spinning disk-coupled confocal microscopy. Z-stack images were acquired using a 491 nm laser for Alexa488 and 561 nm lasers for EM-TAMRA. Acquisition parameters were set at 20% for 561 nm laser and 5% for 491 nm laser and a motor step size of 0.5 μm was used. Images were processed into videos using FIJI (ImageJ, NIH; Bethesda, MD, U.S.A.).
(ZIP)

S8 Fig EM-TAMRA labelling of human primary keratinocytes. Human primary keratinocytes (NHEK) were trypsinised, washed and incubated with 500 nM of EM-TAMRA for 30 min on ice. Labelled cells were subjected to flow cytometry analysis using BD LSRFortessa™. The graph shows 8.58% positive population labelled by EM-TAMRA.
(TIF)

S9 Fig Competition with EM at lower dose range to determine specificity of EM-TAMRA binding to skin cells. (A) Competition with unlabelled Endomorphine-1 at low concentrations (10–250 nM) shows that EM-TAMRA has similar affinities compared to Endomorphine-1. Data were analysed using flow cytometry population analysis. Data displayed are the mean ± SD from three independent experiments. Statistical analysis was performed in GraphPad Prism, version 5.03 using ordinary One-way ANOVA including Dunnett’s multiple comparison *post hoc* test. * P < 0.05; ** P < 0.01; *** P < 0.001.
(TIF)

## Abbreviations

BODIPY: 4,4-Difluoro-4-bora-3a,4a-diaza-s-indacene
BPE: Bovine Pituitary Extract
BTR: BODIPY® Texas Red
cAMP: Cyclic adenosine monophosphate
Ctrl: Control
CTOP: (4R,7S,10S,13R,16S,19R)-N-[(2S,3R)-1-amino-3-hydroxy-1-oxobutan-2-yl]-19-[[(2R)-2-amino-3- phenylpropanoyl]amino]-10-(3-aminopropyl)-7-(1-hydroxyethyl)-16-[(4-hydroxyphenyl)methyl]-13-(1H-indol-3-ylmethyl)-3,3-dimethyl-6,9,12,15,18-pentaoxo-1,2-dithia-5,8,11,14,17-pentazacycloicosane-4-carboxamide
DAMGO: (2S)-2-[[2-[[(2R)-2-[[(2S)-2-amino-3-(4-hydroxyphenyl) propanoyl] amino]propanoyl] amino]acetyl]-methylamino]-N-(2-hydroxyethyl)-3-phenylpropanamide
DIFF: Differentiated
DMF: Dimethylformamide
EGF: Epidermal growth factor
EM: Endomorphine-1
EM-TAMRA: Endomorphine-Tetramethylrhodamine
ER: Endoplasmic reticulum
ESI: Electrospray Ionization
FSK: Forskolin
GPCR: G protein-coupled receptor
HPLC: High-performance liquid chromatography
I: Intensity
Iabs: Absorbance Intensity
IBMX: 1-methyl-3-(2-methylpropyl)-7H-purine-2,6-dione
K-SFM: Keratinocytes serum-free medium
NHEK: Normal human epidermal keratinocytes
NOP: Opioid related nociceptin receptor 1
NTX: Naltrexone, (1S,5R,13R,17S)-4-(cyclopropylmethyl)-10,17-dihydroxy-12-oxa-4-azapentacyclo[9.6.1.0^(61).0^(1).0^(62)]octadeca-7,9,11(18)-trien-14-one
OR: Opioid receptor
PBS: Phosphate buffered saline
PENK: Proenkephalin
PKA: Protein kinase A
POMC: Proopiomelanocortin
RLU: Relative light unit
RT: Room temperature
SD: Standard deviation
SSC-A: Side-scattered area
TAMRA: Tetramethylrhodamine
WGA: Wheat germ agglutinin
δ-OR: Opioid receptor delta 1
κ-OR: Opioid receptor kappa 1
μ-OR: Opioid receptor mu 1
