# Supplementary figures and images for: Investigating endogenous µ-opioid receptors in human keratinocytes as pharmacological targets using novel fluorescent ligand
Source: PLoS One. 2017 Dec 6;12(12):e0188607. doi: 10.1371/journal.pone.0188607 (PMC5718609; doi:10.1371/journal.pone.0188607)

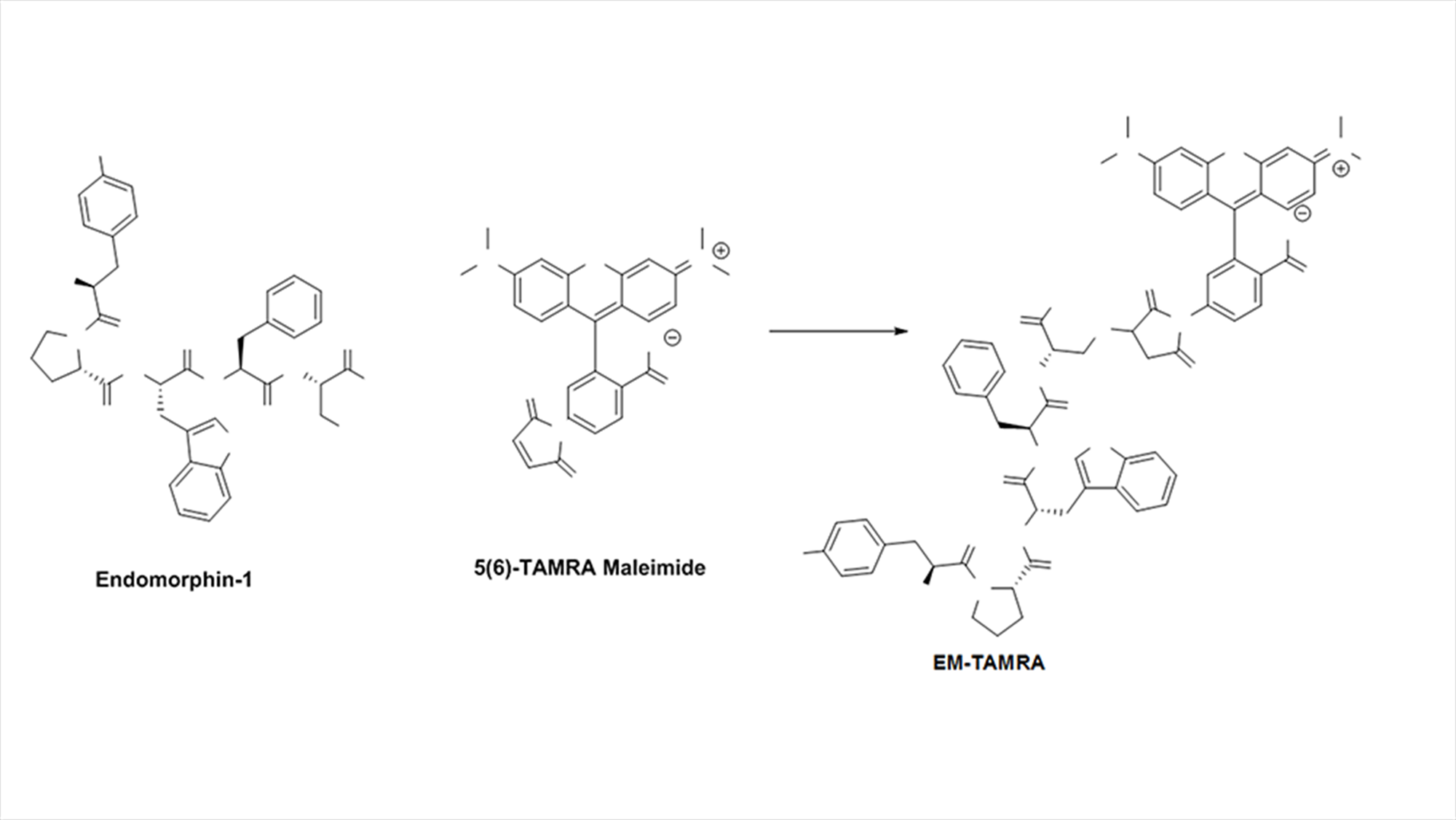

Supplement: S1 Fig — (TIF) [file pone.0188607.s001.tif]

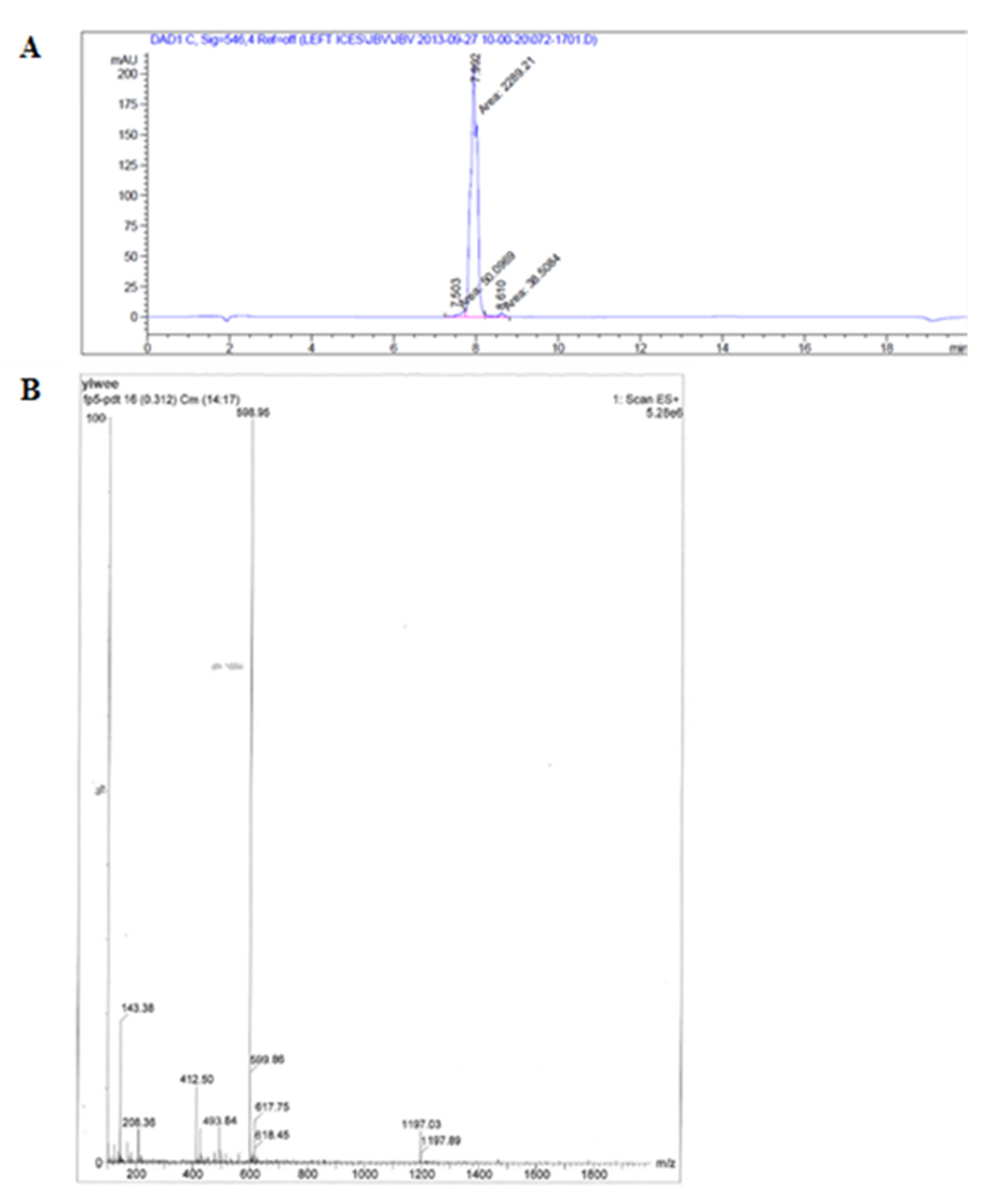

Supplement: S2 Fig — (A) EM-TAMRA was purified using a Jupiter C12 Proteo 90Å RP-HPLC preparative column (10 mm x 250 mm x 4 μm), detector wavelength (220 nm, 546 nm) and following solvent gradients, solvent A (100% H2O + 0.1% TFA) and solvent B (100% acetonitrile + 0.1% TFA). The purity of each fraction were analysed using an analytical HPLC with a Jupiter C12 Proteo 90Å RP-HPLC column (4.6 mm x 150 mm x 4 μm), detector wavelength (220 nm, 546 nm) and following solvent gradients, solvent A (100% H2O + 0.1% TFA) and solvent B (100% acetonitrile + 0.1% TFA). (B) ESI mass of EM-TAMRA was taken using solvent A (100% H2O + 0.1% Formic acid, solvent B (100% acetonitrile + 0.1% formic acid). It was run in isocratic 80:20 (B:A) for two minutes without passing a column. The capillary voltage used during measurement was 3.50 kV. (TIF) [file pone.0188607.s002.tif]

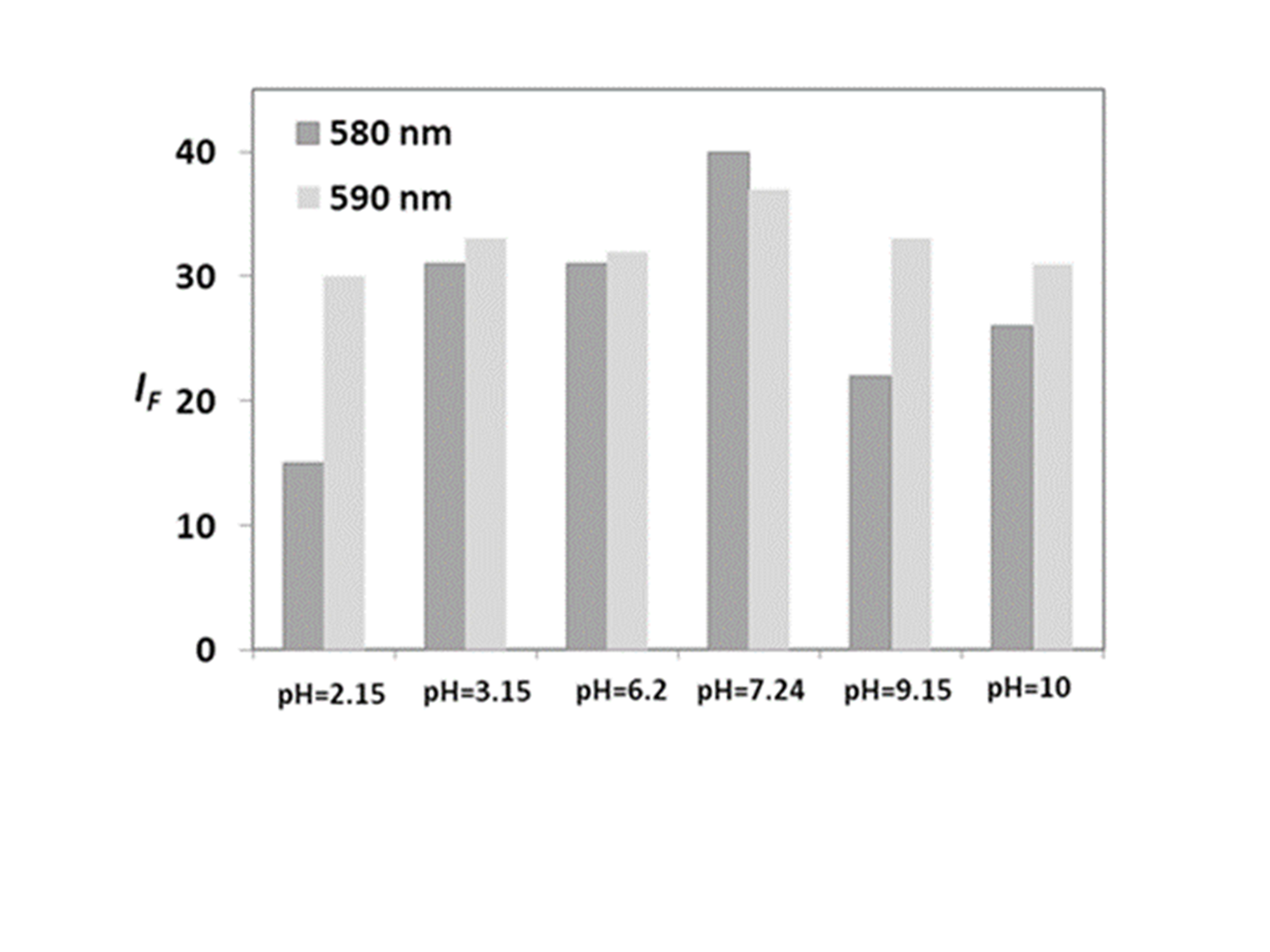

Supplement: S3 Fig — The emission spectra of EM-TAMRA (20 μM) was measured at 580 nm and 590 nm wavelengths for six different pH of K-SFM buffer. λexc = 560 nm. (TIF) [file pone.0188607.s003.tif]

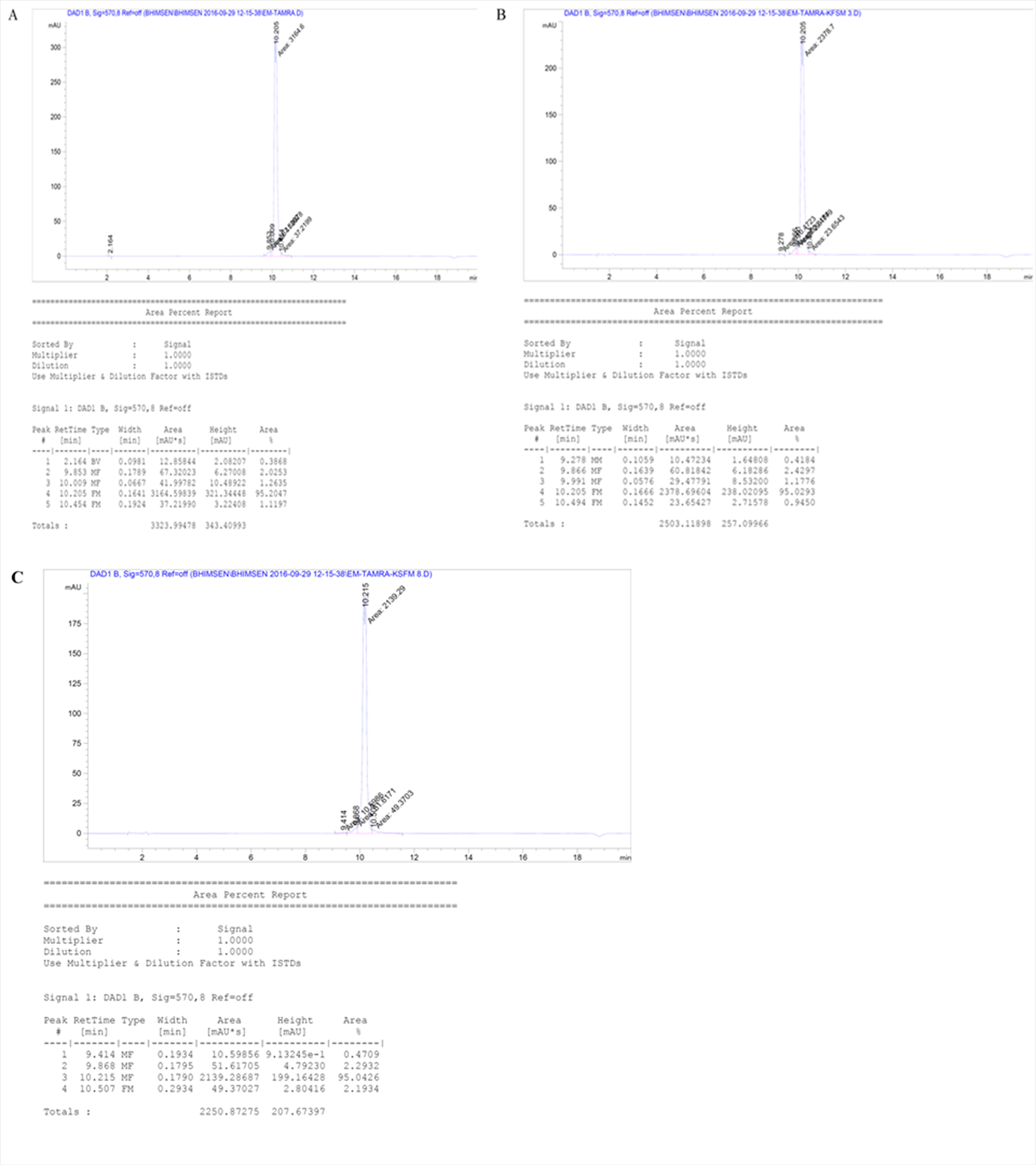

Supplement: S4 Fig — Stability of EM-TAMRA at pH 3.48 (B) and pH 8 (C) in keratinocyte serum-free medium (K-SFM) were studied by analytical HPLC incubating EM-TAMRA for 90 minutes. HPLC of samples were measured using 570 nm to investigate dissociation products. EM-TAMRA is stable under both acidic pH 3.48 (B) and basic pH 8 (C) condition as compared to no K-SFM buffer (A). (TIF) [file pone.0188607.s004.tif]

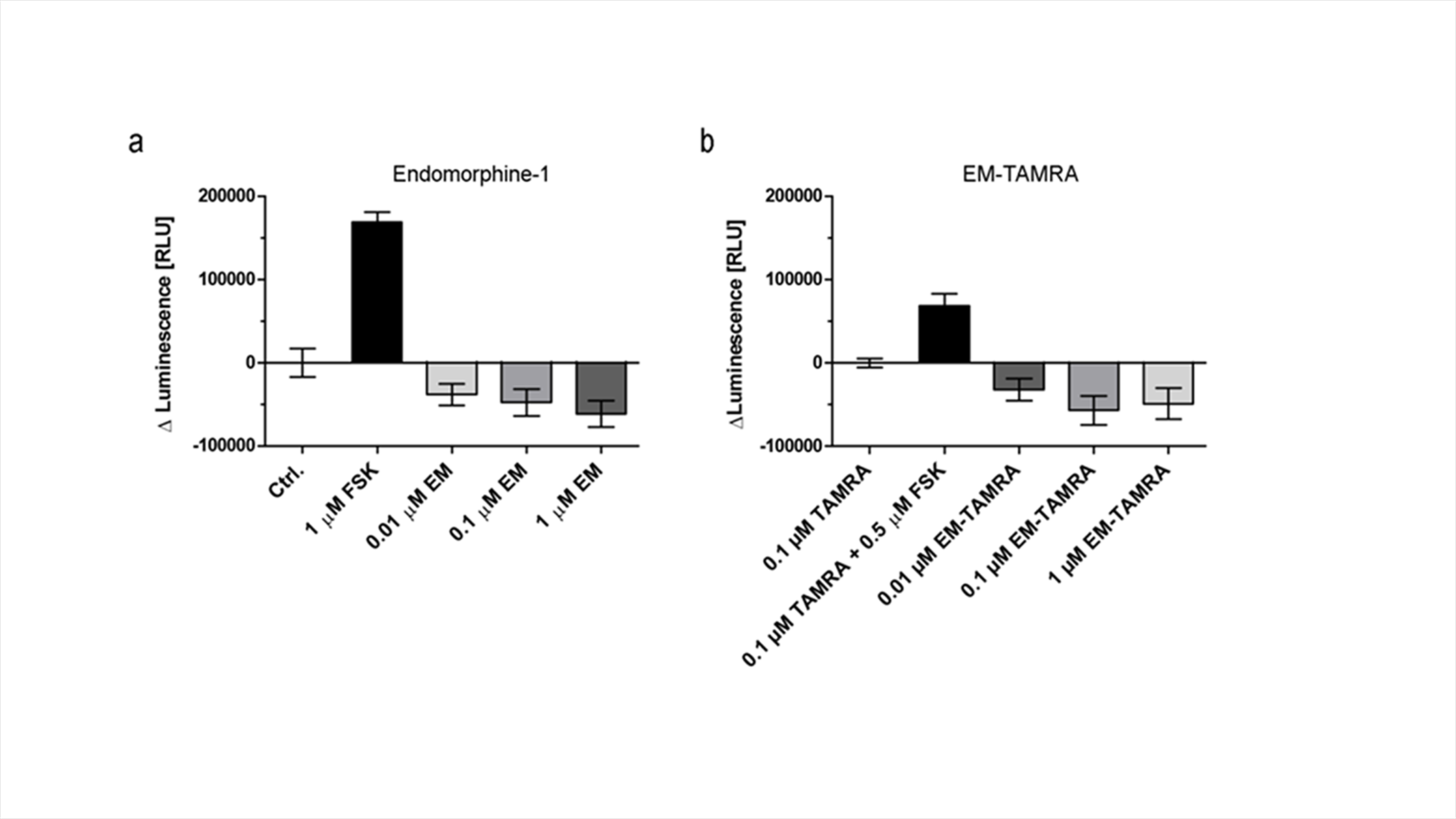

Supplement: S5 Fig — N/TERT-1 keratinocytes were plated in to 96-well plates at 8000 cells/well and grown to 80% confluence. On the day of the cAMP assay the adherent cells were treated with PBS-IBMX buffer (100 μM IBMX + 0.4 mM CaCl2) for 30 min to inactivate phosphodiesterase. The induction buffer (PBS + 20 mM MgCl2) was used to dilute test compounds at different concentrations (agonist, Forskolin and TAMRA control). Cells were treated in 40 μl of induction buffer with relevant test compounds for 30 min at 37°C. 10 μl cAMP detection solution (buffer with enzyme PKA) was added to cells and incubated for 20 min. Cell lysates (50 μl) were transferred into a white-bottom 96-well plate (Greiner Bio-One GMBH, Frickenhausen, Germany). After addition of 50 μl Kinase-Glo reagent reaction was performed for 10 min before measuring luminescence using BioTek Synergy™ H1 plate reader (BioTek; Winooski, VT, U.S.A.). All the procedures were followed according to Promega cAMP-Glo™ Max Assay (Madison, WI, U.S.A.). Inhibition of cAMP production upon opioid receptor activation by Endomorphine-1 or the EM-TAMRA conjugate was analyzed. (A) cAMP level relative to untreated control in Forskolin stimulated or Endomorphine-1 (0.01 μM– 1 μM) treated N/TERT-1 keratinocytes. (B) cAMP level in N/TERT-1 keratinocytes normalized to TAMRA control treated samples. Forskolin stimulation was done in the presence of TAMRA to exclude influence of the dye on the assay reading. EM-TAMRA was added in concentration from 0.01 μM to 1 μM. Data from one representative experiment are represented as mean ± SD from three technical replicates. Ctrl. = untreated control; FSK = Forskolin; EM = Endomorphine-1; RLU = Relative Light Units. (TIF) [file pone.0188607.s005.tif]

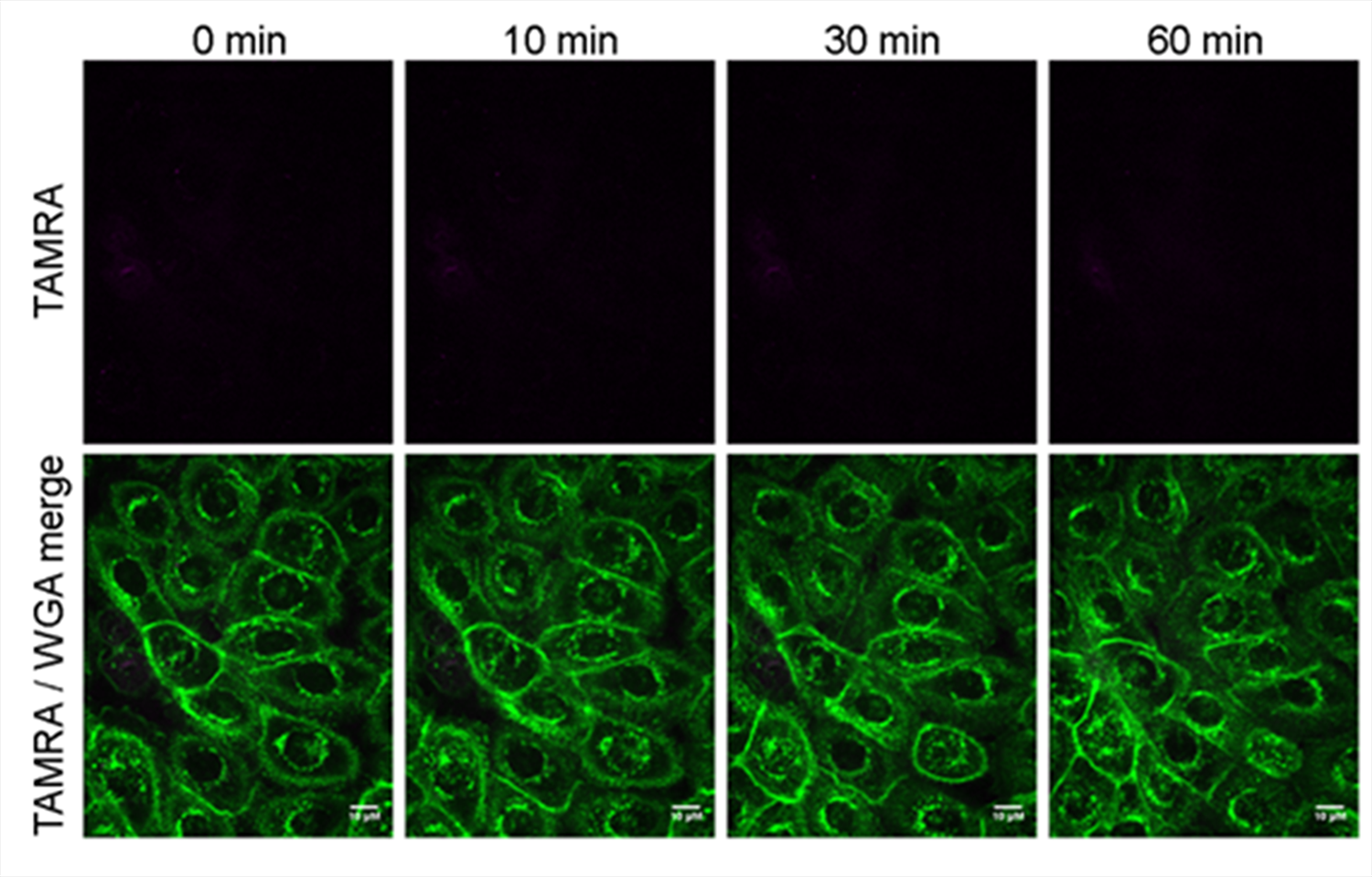

Supplement: S6 Fig — N/TERT-1 keratinocyte membrane and endoplasmic reticulum was labelled for 30 min at 37°C with 5 μg/ml Wheat Germ Agglutinin (WGA Alexa Fluor 488, Thermo Fisher Scientific Inc., Singapore). The cells were washed three times and fresh supplement-free K-SFM was added. TAMRA-Maleimide was diluted in K-SFM containing 0.4 mM CaCl2 in the absence of EGF/BPE. Imaging before binding experiments was carried out to establish the auto-fluorescence of the cells for background adjustments. TAMRA was added at a final concentration of 200 nM and cells visualized by spinning disk-coupled confocal microscopy. Z-stack images were acquired using a 491 nm laser for Alexa488 and 561 nm lasers for TAMRA. Acquisition parameters were set at 20% for 561 nm laser and 5% for 491 nm laser and a motor step size of 0.1 μm was used. Images were analysed using FIJI (ImageJ, NIH; Bethesda, MD, U.S.A.). Weak non-specific staining of keratinocytes by TAMRA can be observed due to the interaction of the dye with lipids of the cell membrane. The staining intensity and pattern does not reflect the staining observed for EM-TAMRA. No internalisation of TAMRA is seen after prolonged incubation over 2 h. EM-TAMRA keratinocyte labelling is therefore caused by the specific interaction of EM with μ-OR cell surface receptors and receptor-mediated internalisation. (TIF) [file pone.0188607.s006.tif]

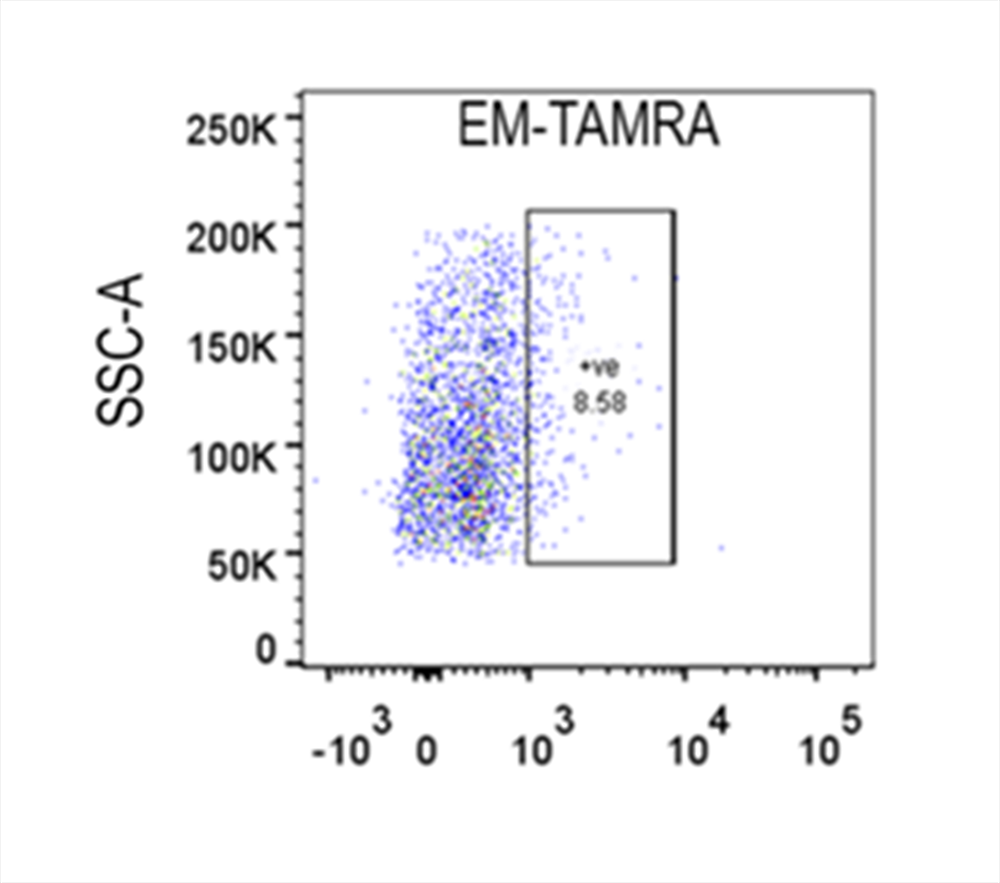

Supplement: S8 Fig — Human primary keratinocytes (NHEK) were trypsinised, washed and incubated with 500 nM of EM-TAMRA for 30 min on ice. Labelled cells were subjected to flow cytometry analysis using BD LSRFortessa™. The graph shows 8.58% positive population labelled by EM-TAMRA. (TIF) [file pone.0188607.s008.tif]

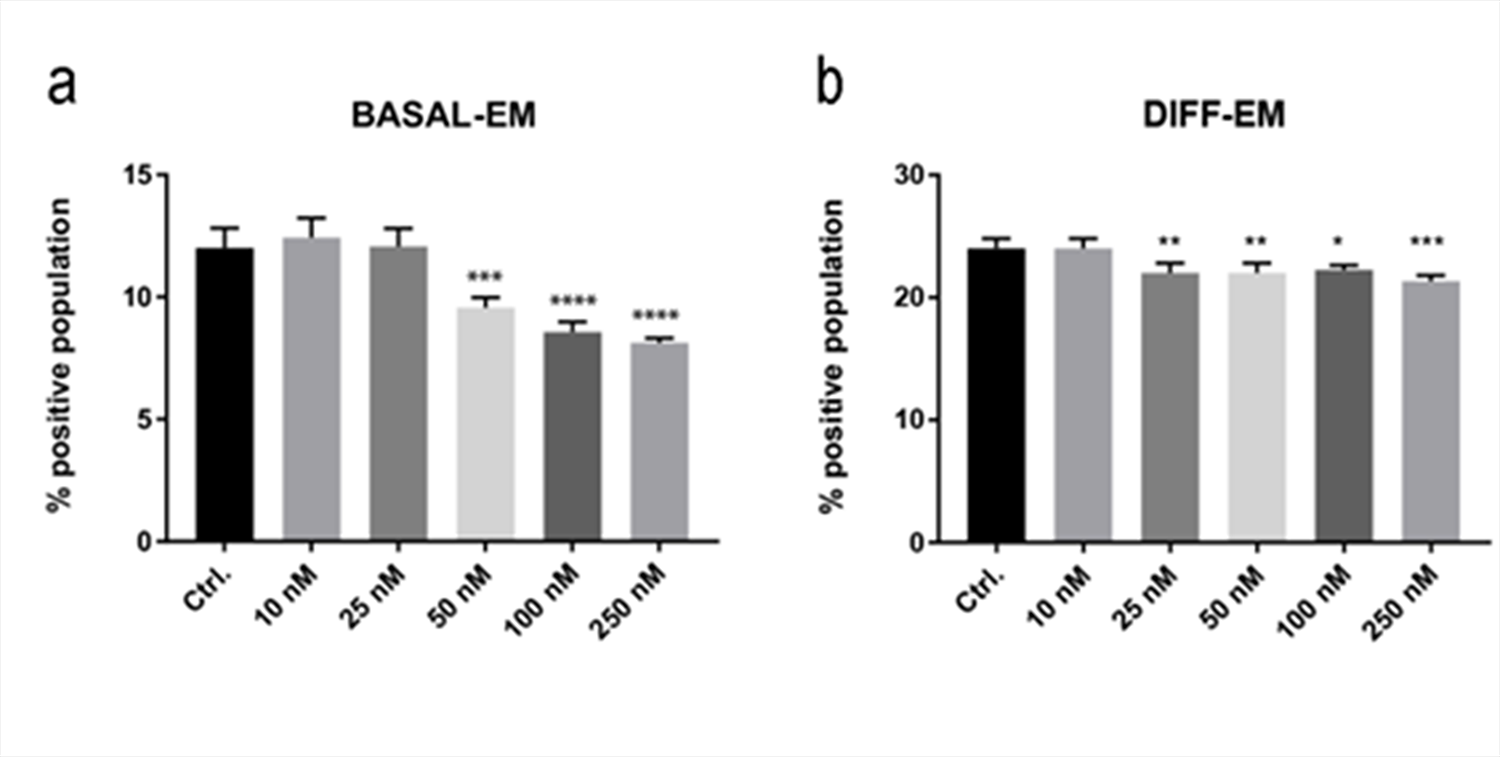

Supplement: S9 Fig — (A) Competition with unlabelled Endomorphine-1 at low concentrations (10–250 nM) shows that EM-TAMRA has similar affinities compared to Endomorphine-1. Data were analysed using flow cytometry population analysis. Data displayed are the mean ± SD from three independent experiments. Statistical analysis was performed in GraphPad Prism, version 5.03 using ordinary One-way ANOVA including Dunnett’s multiple comparison post hoc test. * P < 0.05; ** P < 0.01; *** P < 0.001. (TIF) [file pone.0188607.s009.tif]
